# ACTH-producing carcinoma of the pituitary with refractory Cushing's Disease and hepatic metastases: a case report and review of the literature

**DOI:** 10.1186/1477-7819-7-39

**Published:** 2009-04-08

**Authors:** Scott N Pinchot, Rebecca Sippel, Herbert Chen

**Affiliations:** 1 Section of Endocrine Surgery, Department of Surgery, University of Wisconsin, Madison, WI, USA; 2 H4/750 Clinical Science Center, 600 Highland Avenue, Madison, WI 53792, USA

## Abstract

**Background:**

Pituitary carcinomas are rare neuroendocrine tumors affecting the adenohypophysis. The hallmark of these lesions is the demonstration of distant metastatic spread. To date, few well-documented cases have been reported in the literature.

**Case presentation:**

Here, we report the case of a fatal pituitary carcinoma evolving within two years from an adrenocorticotrophic hormone (ACTH)-secreting macroadenoma and review the global literature regarding this rare neuroendocrine tumor.

**Conclusion:**

Pituitary carcinomas are extremely rare neoplasms, representing only 0.1% to 0.2% of all pituitary tumors. To date, little is understood about the molecular basis of malignant transformation. The latency period between initial presentation of a pituitary adenoma and the development of distal metastases marking carcinoma is extremely variable, and some patients may live well over 10 years with pituitary carcinoma.

## Background

While pituitary tumors represent from 10 to 25% of all intracranial neoplasms, the incidence of pituitary carcinomas is extremely rare[1,2]. In fact, carcinomas account for only 0.1–0.2% of all pituitary neoplasms[3,4]. Like adenomas, the vast majority of reported pituitary carcinomas are endocrinologically active (88%), with most secreting adrenocorticotrophic hormone (ACTH) or prolactin (PRL)[3]. Rarely, growth hormone (GH), leutinizing hormone (LH) and follicle-stimulating hormone (FSH), or thyroid-stimulating hormone (TSH) may be elicited[3]. Histologically, there are no unequivocal findings which distinguish pituitary adenomas from carcinomas; therefore, a diagnosis of pituitary carcinoma depends upon the demonstration of metastatic spread to remote areas of the central nervous system (CNS) or outside the CNS[2,5-7]. Disseminated via the cerebrospinal fluid or by direct parenchymal spread, metastases to the CNS typically invade the brain, spinal cord, and leptomeninges. Less commonly, pituitary carcinomas may metastasize hematogenously – a prominent feature of ACTH-producing carcinomas – resulting in metastatic invasion of the liver, bone, ovaries, heart, and lung. We describe a patient with an ACTH-producing carcinoma of the pituitary with refractory Cushing's disease and hepatic metastases.

## Case presentation

A 59-year-old post-menopausal woman presented to her primary care physician in May 2003 with complaints of fatigue and progressive diplopia. Her past medical history revealed chronic depression and hyperlipidemia but was otherwise negative. Family history was pertinent for a paternal grandfather with thyroid disease and diabetes. When evaluated she weighed 79.0 kg and was 167.6 cm tall (body mass index, 28.1 kg/m^2^). The blood pressure was 136/62 mmHg and the pulse was 88 bpm and regular. Physical examination revealed full extraocular movements, though the patient complained of severe diplopia with extreme right lateral gaze. No neck mass or thyromegaly was present. She did not display typical features of Cushing's syndrome such as moon facies, truncal obesity, buffalo hump, purple striae, skin atrophy, muscle weakness, or hirsuitism.

Hormonal evaluation at the time of admission was significant for a mildly elevated prolactin level of 55.9 ng/mL (normal range 0.4–29.0 ng/mL). Thyroid function tests suggested borderline hypothyroidism (TSH 0.69 μIU/mL (normal range 0.50–4.70 μIU/mL0), free T4 0.8 ng/dL (normal range 0.7–1.8 ng/dL), and total T3 < 30 ng/dL (normal range 45–137 ng/dL)). The morning cortisol was 27.7 μg/dL (normal range 6–24 μg/dL). Serum leutinizing hormone (LH) and follicle-stimulating hormone (FSH) were inconsistent with the patient's post-menopausal status (LH 0.4 mIU/mL (normal range in post-menopause 7.7–58.5 mIU/mL), FSH 3.9 mIU/mL (normal range in post-menopause 25.8–134.8 mIU/mL)).

**Figure 1:**
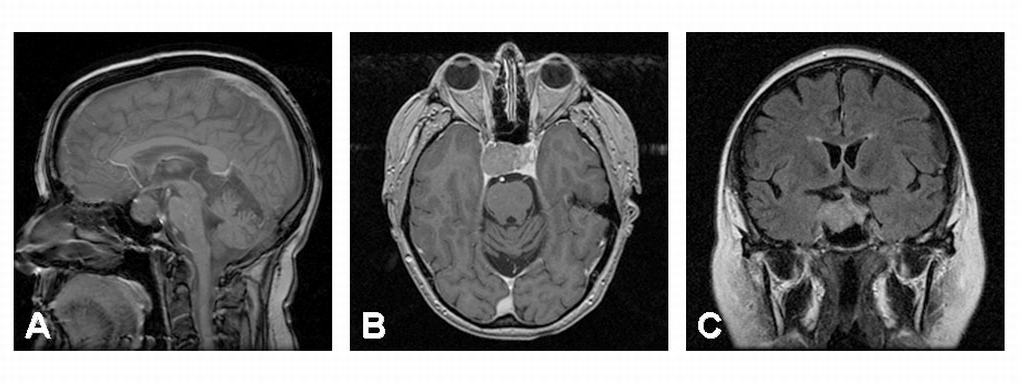
**Pre-operative magnetic resonance imaging (MRI) of the head with and without contrast**. Prior to endoscopic transnasal resection, midsagittal (A), axial (B) and coronal (C) MRI imaging reveal a homogeneous mass involving the sella turcica with extension into the right cavernous sinus which measures 2.8 cm × 2.1 cm × 1.7 cm. The mass does extend up into the suprasellar cistern, but does not impinge upon the optic apparatus. There is partial encasement of the cavernous internal carotid artery on the right side, but the caliber of the vessel is not compromised.

**Figure 2:**
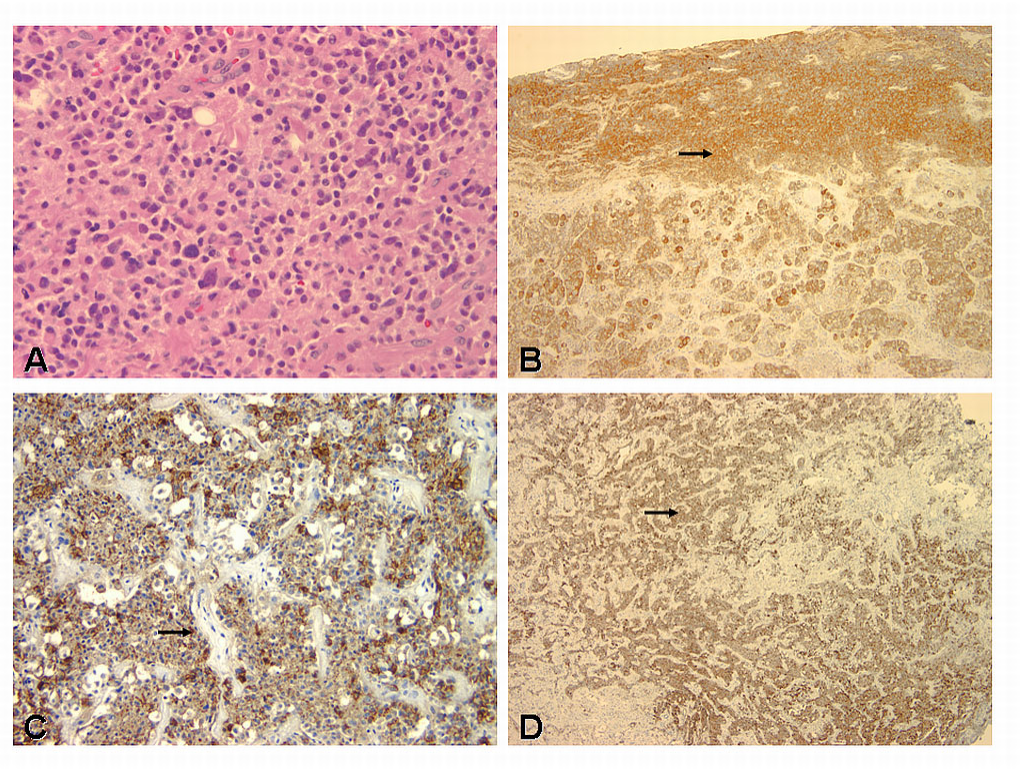
**Photomicrograph comparison of the histological and immunohistochemical features of the adrenocorticotrophic hormone (ACTH)-secreting pituitary tumor and its metastasis to the liver (carcinoma)**. A: Pituitary tumor composed of uniform cells with abundant cytoplasm. Mitotic figures are not observed. B: Tumor showing peripheral cytoplasmic ACTH immunoreactivity. C through D: Pituitary carcinoma with liver metastases. The hepatic nodule shows immunohistochemical reactivity for ACTH and chromogranin A (CgA). H&E (A) and immunoperoxidase staining for ACTH (B and C) and chromogranin A (D). Original magnification × 40 (A), × 10 (B), × 4 (C-D).

With the recent progression of diplopia, magnetic resonance imaging (MRI) of the brain was obtained. This revealed a 2.8 × 2.1 × 1.7 cm homogeneous pituitary mass involving the sella turcica with extension into the right cavernous sinus. Suprasellar extension was noted to the level of the cistern, but no compression of the optic chiasm was apparent. The right cavernous internal carotid artery was partially encased with tumor, but the caliber of the vessel was not compromised (Figure 1). A preliminary diagnosis of pituitary macroadenoma with subsequent partial right sixth nerve palsy was made and the patient was referred for surgery. Using the Stealth frameless stereotactic system, debulking of the tumor by endoscopic transnasal resection was performed in May 2003. Residual tumor was left within the cavernous sinus due to the high risk of cranial nerve injury associated with attempted tumor debulking in this region. The resected tumor was pathologically diagnosed as an adrenocorticotrophic hormone (ACTH) producing pituitary adenoma with extension into the respiratory mucosa (Figure 2). The patient's immediate postoperative course was uncomplicated. She was sent home on oral prednisone. Her serum cortisol levels were monitored and remained normal, and the prednisone dose was tapered.

**Figure 3:**
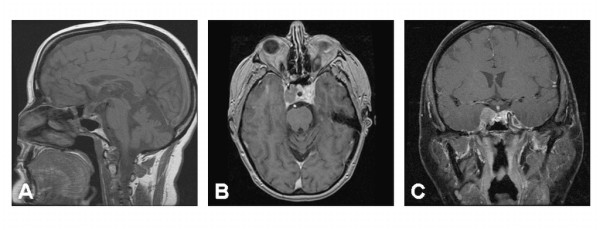
**Post-operative MRI of the head with and withoutcontrast**. Midsagittal (A), axial (B) and coronal (B) imaging suggests an overall interval reduction of the size of the mass within the sella turcica. There is persistent tumor present within the right cavernous sinus. This has maximal measurements of 2.0 cm × 1.3 cm × 0.9 cm. The residual tumor partially encircles the right cavernous internal carotid artery in the same region. There is extension along the posterior cavernous sinus into Meckel's cave and along the dorsal superior clivus adjacent to the basilar artery. Additional extension is seen ventrally along the cranial nerves to abut the posterior optic canal.

MRI imaging of the brain was obtained two months following tumor debulking. A persistent mass was noted within the right cavernous sinus. Measuring 2.0 × 1.3 × 0.9 cm, the residual tumor partially encircled the cavernous right internal carotid artery and extended along the posterior cavernous sinus into Meckel's cave. Ventral extension was noted along the cranial nerves to abut the posterior optic canal (Figure 3). External beam radiation therapy was recommended to address the obvious areas of residual tumor seen on the postoperative MRI. In late 2003, optically-guided fractionated stereotactic radiotherapy was utilized to deliver a dose of 50.4 Gy in 28 fractions to the pituitary with curative intent. Radiotherapy was completed in October 2003. Post-radiotherapy MRI of the brain revealed a marked interval decrease in size of the pituitary mass; furthermore, encasement of the right cavernous internal carotid artery was no longer visualized.

Unfortunately, despite a favorable tumor response to surgical debulking and subsequent radiation therapy, the patient continued to complain of severe fatigue and worsening depression. She was seen by her endocrinologist in November 2003 and a thorough laboratory evaluation was performed. Though serum cortisol levels remained normal, 24-hour urinary free cortisol was elevated at 179.9 μg/day (normal range < 45 μg/day), suggesting pituitary Cushing's disease. She was started on ketoconazole therapy for treatment of these elevated urinary free cortisol levels. Serum calcium was elevated between 10.2–10.7 mg/dL (normal range 8.5–10.2 mg/dL) and serum intact parathyroid hormone (PTH) was mildly elevated at 69 pg/mL (normal range 15–65 pg/mL) suggesting primary hyperparathyroidism. Thyroid function tests showed a high-normal free T4 (1.8 ng/dL, normal range 0.7–1.8 ng/dL) and depressed TSH (0.20 μIU/mL, normal range 0.5–4.7 μIU/mL), reflecting previously started thyroid hormone supplementation. The dose of thyroid supplementation (Synthroid) was decreased in response to these values. She also had a neuroendocrine hormone panel, which showed normal levels of calcitonin, gastrin releasing polypeptide, gastrin, neurotensin, pancreatic polypeptide, VIP, and substance P; however, an isolated elevated pancreastatin level was noted (402 pg/mL, normal range < 135 pg/mL). Multiple endocrine neoplasia type 1 (MEN-1) was considered due to the pituitary, parathyroid, and pancreatic involvement of the patient's endocrinopathies, but genetic diagnostic testing ultimately identified no disease-associated sequence changes on analysis of the MENIN gene.

In December 2003, while awaiting the results from genetic testing, the patient underwent a Tc^99^-sestamibi scan for evaluation of her primary hyperparathyroidism. Parathyroid scans revealed excess radionuclide uptake of sestamibi in the left lower position, suggesting the presence of a parathyroid adenoma. At the same time, MRI of the abdomen was obtained to evaluate for a possible MEN-1 related pancreatic tumor in light of the elevated pancreastatin level. There was no evidence of a pancreatic mass on MRI. The patient was taken to the operating room in March 2004 for a minimally invasive parathyroidectomy. A parathyroid adenoma was identified and removed; intraoperative PTH levels normalized within 10 minutes following removal of the adenoma. Surgical pathology was consistent with the diagnosis of a hypercellular parathyroid gland. Postoperatively, the patient's intact PTH normalized (44 pg/mL, normal range 15–65 pg/mL), but her serum calcium remained slightly elevated (10.3 mg/dL, normal range 8.5–10.2 mg/dL) on oral calcium.

The patient returned to the endocrinology clinic urgently in late September 2004 complaining of severe fatigue, rapid weight gain in excess of 13 pounds over a two week period, facial acne, easy bruisability, tachycardia with exertion and increasing abdominal pain. Urinary free cortisol was found to be severely elevated, measuring 396 μg/day (normal range < 45 μg/day). Ketoconazole therapy was restarted to address the symptoms of Cushing's syndrome. Unfortunately, in November 2004 the patient experienced a bout of diverticulitis with associated sigmoid colon perforation; a sigmoid colectomy was performed with formation of a Hartmann pouch and end colostomy. Her recovery was relatively uneventful.

By December 2004, the patient showed only minimal response to ketoconazole therapy (urinary free cortisol 264.5 μg/day (normal range < 45 μg/day)). A follow-up MRI of the brain revealed a marked increase in size of the pituitary mass to 2.4 × 2.0 × 1.9 cm from 1.1 × 1.0 cm just six months earlier. She additionally noted the rapid development of a complete right 6^th ^cranial nerve palsy. Tumor debulking was again performed in February 2005 via a right frontotemporal orbitozygomatic approach. Final surgical pathology was consistent with an ACTH-producing pituitary macroadenoma. Postoperative stereotactic radiosurgery to the recurrent pituitary tumor was performed to 12.5 Gy. Despite a persistent right-sided ptosis and restricted upward medial gaze with the right eye, the patient noted some improvement in general functional status.

The patient continued to experience symptoms of Cushing's disease despite undergoing a second tumor debulking with subsequent radiotherapy. By spring 2005, she noted severe hypertension and lower extremity edema. Her course was additionally complicated by refractory potassium wasting despite the initiation of potassium-sparing diuretic therapy. The decision to withhold additional ketoconazole treatment was made due to the rapid tumor enlargement noted during the previous trial of the drug. Due to her refractory Cushing's disease, the patient elected to proceed with bilateral adrenalectomy. She additionally requested that her colostomy be taken down at the time of surgery. In August 2005, bilateral open adrenalectomy and colostomy takedown with colorectostomy were performed. Intraoperative examination of the liver revealed a small lesion on the superior right lobe of the liver and a wedge biopsy of this lesion was taken. Final surgical pathology confirmed the presence pituitary carcinoma metastases within the liver parenchyma. Both adrenal glands exhibited cortical hyperplasia. The patient tolerated the procedure well and returned to the post-surgical nursing ward in stable condition.

Postoperatively, the patient's condition declined. Her initial postoperative course was complicated by delayed return of bowel function, severe depression, weakness, and a mild infection at her surgical site. She received perioperative stress dose steroids and was subsequently tapered to an oral dose of prednisone. An octreotide scan, abdominopelvic CT, and brain MRI were ordered at the request of the medical oncology service to detect additional pituitary carcinoma metastases. The octreotide SPECT images of the abdomen and pelvis were within normal limits. The abdominopelvic CT scan revealed multiple hyperenhancing liver lesions most compatible with metastases. Likewise, MRI of the brain showed a small enhancing nodule on the surface of the anteromedial temporal lobe likely representing additional tumor spread. Over the subsequent week the patient's appetite declined and she complained of severe weakness. Steroid boluses were given with little effect. On postoperative day 20 the patient became hypotensive and unarousable. Aggressive fluid resuscitation and cardiopulmonary support with transcutaneous pacing and vasopressor therapy were initiated. She stabilized for a short period but showed little improvement. The decision to withdraw supportive care was ultimately made on postoperative day 21 and the patient died.

## Discussion

Pituitary carcinomas are rare neoplasms of the adenohypophysis, representing only 0.1–0.2% of all pituitary tumors[3,4]. To date, only 150 well-documented cases have been reported in the English literature[3,8-18]. ACTH-producing carcinomas represent 25% to 42% of endocrinologically active pituitary carcinomas[2,3,19]. Because there are no unequivocal histopathologic findings that reliably distinguish pituitary macroadenoma from carcinoma, the diagnosis of malignancy is reserved for primary adenohypophyseal neoplasms with documented craniospinal and/or systemic metastases. A thorough review of the literature reveals pituitary carcinomas display a greater tendency for distant systemic metastases than craniospinal metastases[3]. In fact, the rate of systemic metastases for ACTH-producing pituitary carcinomas is between 57% to 67% [2,3]. A small percent of these tumors (13%) exhibit metastatic spread via both mechanisms[4].

The present case report describes a patient initially diagnosed with a pituitary macroadenoma after initial symptoms resulting from mass effect in and around the sella turcica. The patient eventually developed severe Cushing's disease which was refractory to nearly all medical therapies. Her symptoms were finally treated with bilateral adrenalectomy; however, intraoperative findings of hepatic metastases ultimately resulted in a diagnosis of pituitary carcinoma. This patient is unique because of the relatively abbreviated time interval between the presentation of her sellar adenoma and the manifestation of distant systemic metastases.

### Molecular Pathogenesis

Several theories of pathogenesis for pituitary carcinoma have been proposed, yet little is understood about the molecular basis of malignant transformation. Gaffey *et al. *[20] and others [4,21-23] suggest a progressive adenoma-to-carcinoma sequence based on laboratory observations of histological findings, molecular marker analysis, and a loss-of-heterozygosity analysis between pituitary tumors and their metastases. However, a recent case report undermines this theory by describing the distinct clonal composition of a primary and metastatic ACTH-producing pituitary carcinoma[24].

Studies evaluating the molecular pathogenesis of pituitary carcinoma are ongoing, and several genetic defects have been described. Inactivation of multiple endocrine neoplasia type 1 gene (MEN1) results in the development of multiple endocrine tumors, including pituitary adenomas, in mice and humans. However, this gene does not appear to increase the risk for developing pituitary carcinomas[3]. More recently, Matoso and colleagues[25] have shown that loss of the wild-type retinoblastoma 1 (Rb) gene may lead to MEN-like phenotype in Rb mice. Heterozygous deletions of the Rb gene have been implicated in pituitary carcinogenesis for some time [26,27]. Hinton *et al. *[28] described a patient with two histologically distinct synchronous pituitary lesions; one tumor was a benign ACTH-producing adenoma while the other was an ACTH-producing pituitary carcinoma with distant metastases. The adenoma was found to express the *Rb *gene while the carcinoma displayed no *Rb *genetic expression, indicating the carcinogenic potential of loss-of-function *Rb *mutations.

Similarly, the p53 oncogene has been implicated in pituitary carcinogenesis. In a clinicopathologic study of 15 cases of pituitary carcinoma, Pernicone *et al. *[4] demonstrated an increase in the percentage of nuclear staining for p53 oncoprotein in pituitary metastases (mean, 7.3%) as compared with solitary pituitary adenomas (mean, 1%). Supporting these data, Thapar *et al. *[29] described a significant association between p53 expression and tumor invasiveness, with demonstrated p53 immunohistochemical labeling in 0% of noninvasive adenomas, 15.2% of invasive tumors, and 100% of metastases. These data suggest that p53 expression analysis may be a promising avenue for assessing aggressive tumor behavior.

### Latency Period and Survival

The latency period between the presentation of a sellar adenoma and the development of pituitary carcinoma as manifested by metastatic disease is quite variable. Pernicone *et al*. [4] reported a clinicopathologic study of 15 patients with pituitary carcinomas in which the overall latency period ranged from just over three months to 18 years (median 5 years); the latency was nearly twice as long for ACTH-producing tumors as for prolactin (PRL) tumors (9.5 vs. 4.7 years). In the same series, patients with Nelson's syndrome exhibited the longest mean time interval between adenoma diagnosis and development of metastases (15.3 years) [4]. Similarly, Garrão *et al. *[19] reviewed the cases of several patients with ACTH-producing pituitary carcinomas who had received radiation prior to diagnosis of the carcinoma; in this series, the time interval between the first course of fractionated radiotherapy and the development of metastases varied from 15 months to 18 years. Indeed, while radiotherapy was locally effective in our patient – post-radiotherapy MRI of the brain revealed a marked interval decrease in size of the pituitary mass – the time interval between the presentation of her pituitary macroadenoma and the manifestation of hepatic metastases was only 26 months.

Though several reports of long-term survivors have been published [2,9,23], the long-term prognosis of patients with a diagnosis of pituitary carcinoma is dismal. Pernicone *et al. *[4] noted that nearly 80% of patients died of metastatic disease 7 days to 8 years after diagnosis of any pituitary carcinoma; of these, 66% died within 1 year. More specifically, Landman *et al. *[2] reviewed 33 cases of ACTH-producing pituitary carcinoma, noting that survival from diagnosis of carcinoma to death averaged only 17 months. The shortest reported survival after carcinoma diagnosis was just over 5 weeks while the longest reported survival was 21 years [2]. In the same series, survival was found to be associated with location of metastases; metastases outside the craniospinal axes were associated with a shorter survival time than metastases confined to the CNS (6.6 s vs. 13.7 years) [2]. Unfortunately, our patient survived only 21 days after the discovery of hepatic metastases, representing perhaps one of the shortest time intervals between initial carcinoma diagnosis and death.

### Treatment Modalities

The treatment of choice in pituitary Cushing's disease is transsphenoidal pituitary microsurgery, though other procedures – namely bilateral adrenalectomy and pituitary irradiation – are frequently utilized in cases of refractory disease [30]. Unfortunately, to date there is no curative standard therapy for pituitary carcinoma, and patients are often treated with a combination of the aforementioned procedures in an attempt to palliate symptoms [3]. However, a potential benefit to aggressive therapy has been shown [2,4]. In a series of 15 cases of pituitary carcinoma, Pernicone *et al. *[4] demonstrated a potential survival benefit to aggressive surgical therapy for patients with metastatic deposits within the CNS; in fact, the patient with the longest survival in their case series underwent repeated resections of cerebellar metastases. The same series, however, suggested radiation therapy had only a palliative effect in managing pituitary carcinoma. Our patient was treated with transsphenoidal pituitary microsurgery and subsequent pituitary radiotherapy over 2 years before her metastases were discovered. Interestingly, symptomatic relief from her persistent hypercortisolism was short-lived, necessitating bilateral open adrenalectomy for her refractory pituitary Cushing's disease.

The rates of cure and recurrence of Cushing's disease, and the quality of life after transsphenoidal pituitary surgery are still being investigated. In a single-institution series of 162 patients, Sonino *et al*. [30] sought to characterize the risk factors and long-term outcomes associated with transsphenoidal surgery for pituitary-dependent Cushing's disease. Pituitary surgery was successful in alleviating symptoms associated with hypercortisolism in nearly 77% of patients; failure was associated with lack of pituitary adenoma and the clinical severity of pre-operative disease [30]. Unfortunately, the estimated cumulative percentage of patients remaining in remission declined over time (93.7% after 2 yr, 80.6% after 5 yr, 78.5% after 7 yr, 74.1% after 10 yr). A second study by Locatelli *et al. *[31] supports these data, suggesting that 10% to 30% of patients will fail to achieve long-term remission of their Cushing's disease following transsphenoidal surgery.

Bilateral adrenalectomy is occasionally indicated for patients with pituitary Cushing's disease who fail transsphenoidal surgery or radiation therapy. While adrenalectomy may be noted as the surest means of reducing cortisol production in severe forms of Cushing's disease, it may lead to Nelson's syndrome (NS). Originally described by Nelson *et al. *[32] in 1958, NS is defined by the association of a pituitary macroadenoma and high plasma ACTH concentrations following bilateral adrenalectomy. Thought to be due to enhancement of pituitary tumor growth after adrenalectomy, NS may cause debilitating symptoms including visual changes from mass effect and skin hyperpigmentation. The prevalence of NS varies, although many of the largest series suggest a rate between 8% and 29%, with a time interval between adrenalectomy and NS diagnosis of 0.5 to 24 years [33]. These resulting tumors may be locally invasive and have been associated with the development of pituitary apoplexy. Combined modalities – namely pituitary radiation in conjunction with adrenalectomy – may lower the incidence of Nelson's syndrome [34]. In a retrospective review of 39 patients treated by bilateral laparoscopic adrenalectomy for Cushing's disease after transsphenoidal pituitary tumor resection, Thompson *et al. *[35] sought to determine the safety, efficacy, and long-term quality of life after bilateral adrenalectomy for persistent disease. Based on their observations, laparoscopic adrenalectomy was found to be a safe and effective treatment option (zero operative mortalities, 10.3% morbidity rate, 89% of patients noted improvement in Cushing's-related symptoms) [35,36]. The incidence of NS requiring clinical intervention was 8.3%. Based on these data and quality of life assessments, bilateral adrenalectomy was felt to be an adequate alternative treatment modality for severe refractory disease.

## Conclusion

Pituitary carcinomas are extremely rare neoplasms, representing only 0.1% to 0.2% of all pituitary tumors. To date, little is understood about the molecular basis of malignant transformation. The latency period between initial presentation of a pituitary adenoma and the development of distal metastases marking carcinoma is extremely variable, and some patients may live well over 10 years with pituitary carcinoma. We describe a unique patient who unfortunately died relatively quickly from an ACTH-producing pituitary carcinoma. Despite aggressive surgical debulking of the primary disease and subsequent pituitary radiotherapy and bilateral adrenalectomy for control of refractory Cushing's disease, the patient ultimately was found to have hepatic metastases indicating the development of carcinoma. She ultimately died from her disease just 1 month following the diagnosis of carcinoma.

## Competing interests

The authors declare that they have no competing interests.

## Authors' contributions

HC performed the bilateral adrenalectomy and together with SP and RS contributed to the conception and design of the manuscript, reanalyzed and interpreted the data and prepared the manuscript. SP contributed to the conception and design of the manuscript, reanalyzed and interpreted the data and helped in preparation of manuscript. RS. contributed to the conception and design of the manuscript, reanalyzed and interpreted the data. All authors read and approved the manuscript.

## Consent

Written consent from the patient was obtained for publication of this case as part of IRB approved research study. The copy of the consent is available with Editor-in-Chief.
